# Thyroid Dysfunction and Bipolar Disorder: A Literature Review Integrating Neurochemical, Endocrine, and Genetic Perspectives

**DOI:** 10.7759/cureus.69182

**Published:** 2024-09-11

**Authors:** Sarah J Norman, Ayzia C Carney, Fernanda Algarin, Brittany Witt, Ivette M Witzel, Paula M Rodriguez, Moiud Mohyeldin

**Affiliations:** 1 Internal Medicine, American University of the Caribbean School of Medicine, Cupecoy, SXM; 2 Emergency Medicine, American University of the Caribbean School of Medicine, Cupecoy, SXM; 3 Obstetrics and Gynecology, American University of the Caribbean School of Medicine, Cupecoy, SXM; 4 Psychiatry and Behavioral Sciences, American University of the Caribbean School of Medicine, Cupecoy, SXM; 5 Internal Medicine, BronxCare Health System, Bronx, USA

**Keywords:** bipolar affective disorder, bipolar depression, genetics, hpt axis, hyperthyroidism, hypothyroidism, mania, mood disorder, thyroid hormones, thyroid pathology

## Abstract

Thyroid disorders are common in medicine, while bipolar disorders (BDs), though less frequent, are significant due to global prevalence, the economic burden on healthcare systems and long-term health implications, and the effects of psychiatric illness on quality of life. Clinical research has suggested thyroid hormone imbalances can cause psychiatric symptoms similar to the clinical features observed in BDs. Despite increased attention in this area of study, much remains unknown regarding how thyroid issues contribute to the development of BDs. This review explores the complex link between thyroid disorders and BDs, focusing on neurochemical dynamics, changes in the hypothalamic-pituitary-thyroid (HPT) axis, and genetic factors. Furthermore, this literature review examines the importance of understanding these factors in linking both conditions and emphasizes the necessity for therapies targeting their shared underlying mechanisms.

## Introduction and background

Bipolar disorder overview

Bipolar disorder (BD) is a complex psychiatric condition of unclear etiology causing a variable degree of functional impairment. It is defined by cyclic episodes of mood disturbances that span the entire spectrum from mania or hypomania to depression. Given the prevalence and an average diagnostic delay of 9.5 years for BD, it is probable that most clinicians will encounter patients whose management is complicated by this particular psychiatric disorder [1]. Furthermore, BD is strongly associated with higher rates of other psychiatric and physical health comorbidities including substance use disorder, psychiatric medication-induced metabolic disorders such as diabetes and hyperlipidemia, and cardiovascular disease [1]. Although genetic predisposition contributes 60-85% of the risk for the development of BD, other risk factors such as a history of child abuse, excessive cannabis use, and postpartum hormonal changes have also been supported [1].

**Figure 1:**
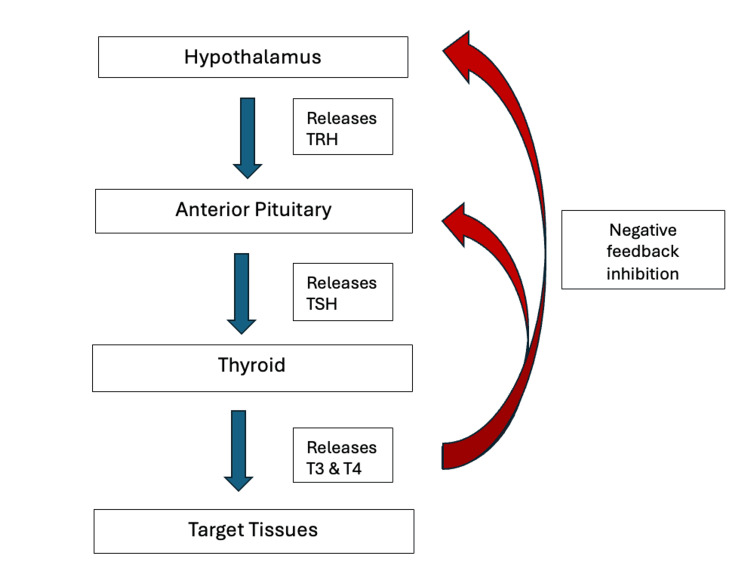
The HPT Axis and Negative Feedback Loop

According to the DSM-V-TR, there are specific criteria that must be met to diagnose BD Type 1 and Type 2. BD Type 1 necessitates the documented history of at least one manic episode, while BD Type 2 warrants at least one hypomanic episode and at least one depressive episode [2]. Table 1 states the DSM-V-TR criteria needed to diagnose each disorder and the episodes associated with BD Type 1 and Type 2. While the hallmark for diagnosing BD Type 1 includes the presence of at least one manic episode, these individuals may also experience hypomania, depressive episodes, and periods of neutral mood [2]. For BD Type 2, patients often seek treatment for their depressive episodes, which then leads to the diagnosis. It is important to note that individuals who experience hypomania tend to be ego-syntonic, meaning an individual's behaviors align with one's personal beliefs, which may cause difficulty in identifying differences in behavior from baseline [3]. Therefore, it is crucial to obtain a thorough history and consider additional information provided by friends and family close to the individual if available [3]. If a hypomanic episode occurs during depressive treatment and meets the criteria from Figure 1, a BD Type 2 diagnosis may be established with caution [3]. In addition to BD Type 1 and BD Type 2, some individuals may be diagnosed with cyclothymic disorder, which is characterized by a two-year period of symptoms of hypomanic and depressive episodes but does not fully meet the criteria of either one [3]. Cyclothymic disorder oftentimes is considered less severe than BD Type 1 and Type 2 and has been successfully treated in some cases with psychotherapy without the need for medication [3].

**Table 1:** BD Type 1 and Type 2 Diagnostic Criteria Table adapted from the DSM-V-TR [2]. BD, bipolar disorder

Criteria	Bipolar Type 1 Disorder	Bipolar Type 2 Disorder
Criteria A: Manic Episode	Presence of at least one manic episode	Presence of at least one hypomanic episode
Criteria B: Depressive Episode	May or may not have experienced a depressive episode	Presence of at least one major depressive episode
Duration	Manic episode lasts 7 or more days	Hypomanic episode lasts 4 or more days
Impairment	Significant impairment in function	May experience significant impairment
Psychosis	Manic episodes may include psychotic features	Hypomanic episodes generally do not include psychotic features
Severity	Symptoms are severe enough to cause marked impairment and can affect occupational and social functioning	Symptoms can cause significant impairment given burden of depression

Pharmacological treatment of BD is complex and continues to be updated in accordance with the ongoing research investigating the long-term effects of the most commonly prescribed medications. Furthermore, pharmaceutical therapy differs with respect to acute bipolar depression, acute bipolar mania, and maintenance therapy. Bipolar depression is typically treated with quetiapine monotherapy or an antidepressant in conjunction with a mood stabilizer (i.e., olanzapine and fluoxetine) [3]. Antidepressant monotherapy for BD Type 1 is discouraged due to the increased emergence of manic symptoms and rapid cycling [4]. However, there is no evidentiary support to avoid antidepressant monotherapy for BD Type 2 or dispute its effectiveness in treating depressive episodes [4]. For initial treatment of acute, severe mania, guidelines include the use of antipsychotics such as aripiprazole, haloperidol, olanzapine, quetiapine, or risperidone in addition to lithium or valproate [3]. Hypomania and mild to moderate mania are treated with antipsychotic monotherapy, typically risperidone, olanzapine, or any of the other alternatives previously mentioned [3]. Electroconvulsive therapy is also a treatment option reserved for patients with severe mania who are refractive to pharmacotherapy [5]. In the setting of maintenance therapy, recommendations continue to include lithium and quetiapine as first-line medications due to their ability to prevent relapses of mood episodes [5].

Hypothalamic-pituitary-thyroid axis overview

The pituitary gland, often referred to as the “master” endocrine gland, produces a variety of hormones that have a downstream effect on the growth, development, and function of their target tissue. Thyroid-releasing hormone (TRH) is released from the hypothalamus, triggering thyroid-stimulating hormone (TSH), which acts directly on the thyroid, prompting the synthesis and release of thyroid hormones thyroxine (T4) and triiodothyronine (T3) [6]. Both T4 and T3 play pivotal roles in regulating growth and metabolism. This regulatory cascade operates through a negative feedback loop: elevated T3 and T4 levels inhibit TRH secretion at the hypothalamic level and suppress TSH production at the anterior pituitary level [6]. Conversely, when thyroid hormones are decreased, they are unable to inhibit the hypothalamic-pituitary-thyroid (HPT) axis, and TRH and TSH are secreted, respectively [6]. Disruptions to this feedback loop, as observed in Graves disease, can lead to excessive thyroid hormone production due to TSH receptor autoantibodies prompting a pathological surge in metabolism [6]. Symptoms of hyperthyroidism include palpitations, weight loss, heat intolerance, fine tremors, and mood disturbances such as irritability and insomnia whereas hypothyroidism is associated with weight gain, cold intolerance, somnolence, impaired cognition, and depressed mood due to a deficiency of thyroid hormones [6]. Treatment for both conditions is aimed at achieving a euthyroid state. For instance, management for hyperthyroidism includes anti-thyroid medication such as propylthiouracil and methimazole [7]. In the setting of recurrent hyperthyroidism, definitive management such as thyroidectomy or radioiodine ablation with subsequent thyroid hormone replacement may be discussed as treatment options. Hypothyroidism is commonly treated with synthetic T4 substitution-levothyroxine with or without liothyronine (synthetic T3) [7].

This current narrative review is not a systematic review, but a narrative review intended to provide a comprehensive outline regarding the association between BD and thyroid dysfunction in the setting of the expansive fields of psychiatry, neurology, endocrinology, and genetics. A systematic review was not conducted, as the authors focused on relevant studies. While this method has a concern for bias, the writers attempted to present the information in this review objectively, while also noting differences in results and study conclusions across the referenced articles. 

Methods

The search strategy included medical subject headings (MeSH) and keywords related to the HPT axis, thyroid dysfunction, BD, and mood disorders using PubMed and Cochrane Library databases. Exclusion criteria included pregnant subjects, and studies published prior to the year 2000, as well as articles published in any language other than English. Inclusion criteria were male and female subjects of all ages, animal studies, meta-analyses, randomized controlled trials (RCTs), cohort studies, case-control studies, and other literature reviews published within the last 20 years.

## Review

Neurochemical Dynamics

Associations Between Neurotransmitters and Bipolar Disorder

Mood disorders are intricate conditions that are influenced by a multitude of factors including various neurotransmitter systems. Several neurotransmitters have been identified as playing significant roles in the pathogenesis of mood disorders, including norepinephrine (NE), serotonin (5-hydroxytryptamine also noted as 5-HT), dopamine (DA), gamma-aminobutyric acid (GABA), and glutamate [8-15]. Interpreting the function of these neurochemicals is essential for elucidating the underlying mechanisms and developing treatments for those with BD.

NE exerts its effects on mood regulation, stress responses, cognitive function, and sleep regulation. Moreover, NE closely interacts with the 5-HT and DA systems, both of which are implicated in mood disorders [8]. NE works synergistically with DA and 5-HT affecting their release and reuptake to modulate mood [9]. NE is also commonly utilized in stress responses, and dysregulation of the stress response can lead to alterations in DA and 5-HT levels further contributing to the genesis of mood disorders [10]. The role of NE in the development and exacerbation of mood disorders is bolstered by the observation that antidepressant treatments, such as norepinephrine reuptake inhibitors (NRIs) and norepinephrine-dopamine reuptake inhibitors (NDRIs), effectively increase synaptic levels of NE alleviating symptoms of affective disorders [10].

5-HT is understood to modulate mood, sleep, appetite, and stress responses. It is hypothesized that 5-HT dysregulation can induce depressive and anxious symptoms, as well as cause emotional dysregulation observed in those with BD [11]. For instance, dysregulation of 5-HT signaling may contribute to difficulties in emotional regulation such as causing heightened emotional reactivity and mood instability during manic episodes [11]. Decreased 5-HT levels have also been linked to impulsivity and aggression observed during manic states [12]. Consequently, the use of SSRIs without concomitant mood stabilizers or antipsychotic medications is typically avoided in BD due to the risk of antidepressant-induced mania or hypomania, which may accelerate cycling between mood episodes and exacerbate manic symptoms [12].

DA influences mood regulation, motivation, reward processing, and movement control. For BD specifically, dysregulation of the DA system can cause mood abnormalities such as impulsivity, mood swings, and states of psychosis [13]. Low levels of DA are thought to contribute to depressive symptoms like anhedonia, low motivation, and fatigue [13]. Conversely, during manic episodes, an increase in DA can lead to activating symptoms such as elevated mood, increased energy, impulsivity, and decreased need for sleep [13]. Medications that modulate DA, such as first-generation antipsychotics, are commonly used to help manage the manic symptoms of BD by blocking DA receptors in the brain [13].

In addition to NE, 5-HT, and DA, alterations in GABAergic neurotransmission are also believed to play a critical role in the pathophysiology of mood disorders [14]. GABA is an inhibitory neurotransmitter that helps regulate the excitability of neurons in the central nervous system. For instance, insufficient GABA activity leads to increased neuronal excitability, contributing to clinical symptoms like agitation and anxiety [14]. Enhancing GABA, like utilizing benzodiazepines or certain antidepressants, helps to alleviate symptoms of anxiety and promote relaxation [14]. Glutamate, a major excitatory neurotransmitter, is understood to have a role in the pathogenesis of BD. For instance, rodent studies have indicated that mood stabilizers impact glutamatergic receptors, influencing mood-related behaviors [15]. Moreover, post-mortem examinations of patients with BD reveal frontal cortex excitotoxicity, altered glutamatergic function, and abnormal synaptic connections [15]. It is important to note that glutamate has an excitatory and inhibitory subtype, which can further complicate its role in psychiatric and neurological disorders [15]. The interplay between NE, 5-HT, and other neurotransmitter systems such as DA, GABA, and glutamate is essential for maintaining euthymia [11]. Any dysregulation in these systems can contribute to the development and exacerbation of mood disorders such as BD. The functions, site of synthesis, and augmenting substances that affect these neurotransmitters are summarized in Table 2.

**Table 2:** Summary of Function, Site of Synthesis, and Augmenting Substances of Neurotransmitters Implicated in BD This table summarizes the functions, sites of synthesis, and augmenting substances of neurotransmitters implicated in BD, adapted from various scholarly sources [8-15]. BD, bipolar disorder; NE, norepinephrine; 5-HT, 5-hydroxytryptamine; GABA, gamma-aminobutyric acid; DA, dopamine

Summary of Function, Site of Synthesis, and Augmenting Substances of Neurotransmitters Implicated in BD			
	Function	Site(s) of synthesis	Augmenting medication/substances
NE	Major NT of the sympathetic nervous system; regulates mood, arousal, attention, memory, and vascular sympathetic tone	Locus coeruleus	Stimulants, cocaine, bupropion, mirtazapine, SNRIs, TCAs
Serotonin (5-HT)	Responsible for modulating mood, sleep, appetite, and stress responses	Raphe nucleus	SSRIs, mirtazapine, and other atypical antidepressants, SNRIs, TCAs, serotonin antagonists, and reuptake inhibitors
GABA	Inhibitory neurotransmitter that helps regulate the excitability of neurons in the brain	Nucleus accumbens	Alcohol, benzodiazepines, phenobarbital, non-benzodiazepine hypnotics
DA	Responsible for mood regulation, motivation, reward processing, and movement	Substantia nigra, ventral tegmental area, hypothalamus	Some antipsychotics, bupropion, NMDAr antagonists, MAO-B inhibitors, COMT inhibitors, DA agonists, decarboxylase inhibitors, DA precursors
Glutamate	Excitatory and inhibitory neurotransmitter involved in memory, executive function, and mood regulation	Glial cells	Alcohol, PCP, cocaine, nicotine

Thyroid Function in the Pathophysiology of Mood Regulation in Affective Disorders

Thyroid function is essential in mood regulation as alterations in thyroid hormone levels can have profound effects on mood and cognition. The thyroid hormones T3 and T4 influence the synthesis, release, and metabolism of neurotransmitters NE, DA, and 5-HT [16]. Alterations in thyroid hormones can disrupt the balance of these neurotransmitters, thereby causing mood dysregulation [17]. For example, thyroid hormones influence the synthesis and bioavailability of tryptophan leading to alterations in 5-HT, which may contribute to emotional dysregulation [17]. Thyroid hormones also have the capacity to regulate particular enzymes, such as tyrosine hydroxylase, involved in DA synthesis and expression [18]. Additionally, thyroid hormones regulate the expression, availability, and activity of 5-HT, DA, and NE transporters affecting their synaptic concentrations and signaling [18]. Likewise, dysregulation in thyroid function can lead to abnormalities in neurotransmitter receptor density and affinity affecting the downstream signaling pathways and generating physiological responses of these chemicals [17]. In addition to affecting neurotransmitters, thyroid hormones play a key role in the brain’s function and development. Thyroid hormone receptors are present throughout the brain including the prefrontal cortex, amygdala, and hippocampal structures [19]. T3 and T4 have also been found to play a factor in neuroplasticity, specifically the ability of the brain to adapt and reorganize its neuronal network [17]. Dysregulation of thyroid hormones can cause functional changes in the brain, affecting cognitive ability, emotional processing, and mood regulation contributing to the severity of bipolar symptoms [19]. 

Several studies have supported the bidirectional relationship between thyroid dysfunction and the development of BD. For example, research by Fagiolini found that 15.3% of patients with BD on lithium carbonate treatment had hypothyroidism, with longer lithium treatment associated with a higher risk of developing thyroid dysfunction [20]. These results emphasize the importance of thyroid monitoring in patients with BD Type 1 undergoing treatment with lithium [20]. Bocchetta's case series linked BD with elevated antithyroid antibodies, suggesting a potential independent vulnerability to thyroid autoimmunity in offspring of those with BD [21]. Treatment with antipsychotics, mood stabilizers, and immunomodulatory therapy improved psychiatric symptoms, normalized thyroid function, and reduced antibody levels [21]. Another study uncovered a high prevalence of thyroid issues in patients with BD, particularly hypothyroidism, ranging from 10% to 30% of the study population [22]. The researchers explored potential mechanisms for this relationship such as neuroendocrine dysregulation and immune-mediated processes such as autoimmune thyroiditis, feasibly linking thyroid dysfunction and BD [22]. Furthermore, a systematic review found that there was an increase in the prevalence of thyroid autoantibodies in patients with bipolar depression in comparison to the general population and suggested thyroid dysfunction to be a risk factor in the development of BD [23]. A study conducted by Ittermann et al. used data from 2142 individuals who participated in the Study of Health in Pomerania Study to determine the association between thyroid disorders and anxiety and depression using the Beck depression inventory (BDI) [24]. The study found hypothyroidism to be favorably linked to an increased BDI score, while hyperthyroidism was found to be positively associated with major depressive disorder (MDD) [24]. Overall, these studies have aimed to uncover the relationship between thyroid disorders and their effect on mood stability in BD as well as depression and anxiety.

Bridging Thyroid Dysfunction and Bipolar Disorder

In the context of investigating the interplay between thyroid dysfunction and BD, it is crucial to discern potential converging pathways in the chemical and physiological mechanisms underlying both conditions. Notably, thyroid hormone, NE, and DA share a common precursor, tyrosine, implying structural and functional similarities among these neurochemicals [22]. Likewise, receptors for neurotransmitter systems and thyroid hormones are localized throughout the limbic system, underscoring the importance of maintaining a balance among these chemical mediators for their regulatory functions [25]. Historically, BD has been associated with disturbances in neurotransmitter balance, characterized by heightened DA and glutamate activity alongside diminished cholinergic muscarinic activity [26]. Post-mortem investigations of BD brains have uncovered globally elevated NE metabolic rates and decreased 5-HIAA rates, despite the absence of concentration irregularities in NE, DA, and serotonin (5-HT) within the cortex and thalamus [27]. These findings align with observed bipolar mood fluctuations, correlating heightened NE levels with manic symptoms and reduced 5-HIAA levels with depressive episodes. In addition, an analysis investigating cerebrospinal fluid (CSF) samples from hypothyroid individuals measured levels of homovanillic acid (HVA), a DA metabolite, and 5-HIAA, a serotonin metabolite and assessed thyroid function [25]. This data was compared with the blood levels of TSH and T3 in the same subjects, revealing a negative correlation between HVA or 5-HIAA levels and TSH/T3 levels [25].

Seeking to comprehend the connection between BD and thyroid dysfunction requires establishing the connection of how thyroid hormone affects neurotransmitter concentration, and how this concentration manifests in BD symptoms. Bauer's research indicates that thyroid hormone infusions elevated concentrations of 5-HT, 5-HTP, and 5-HIAA in the rodent brain cortex, suggesting a desensitizing impact on 5-HT1A raphe presynaptic autoreceptors and a sensitizing effect on cortical 5-HT2 receptors, thereby increasing 5-HT release [28]. Moreover, Bauer noted thyroid hormones' influence on dopaminergic signal transduction [28,29]. A cross-sectional study examining thyroid function in patients with BD analyzed blood samples for TSH, T3, and T4 levels [16]. Elevated T3 levels were significantly correlated with BD, particularly during manic episodes [16]. The authors concluded that individuals with bipolar affective disorder had a 2.5-fold higher risk of impaired thyroid function compared to the general population [16]. Another study by Feng analyzed untreated patients with MDD and BD, measuring thyroid hormone concentration [30]. While 12 of 83 patients showed reduced secretion, analysis configured no significant differences were found in hormone levels between MDD and BD groups [30]. The aforementioned studies emphasize the potential link between thyroid hormones and neurotransmitter imbalances; however, further research is needed to provide a more comprehensive perspective on the development of BD and its manifestations. 

Hypothalamic-pituitary-thyroid axis dysfunction: catalyst for bipolar disorder

Stress Response Systems

The HPT axis interacts directly with the central stress response system. Stress signals are received by the hypothalamus stimulating the release of corticotropin-releasing hormone (CRH), which prompts the pituitary gland to secrete adrenocorticotropic hormone (ACTH) [31]. ACTH then stimulates the adrenal glands to release cortisol [32]. The release of NE and epinephrine from the adrenal medulla, triggered by sympathetic nervous system activation, can further stimulate the release of cortisol as part of the stress response [31].

Understanding cortisol and its effects on the HPT axis may assist investigators in further understanding the associations between thyroid dysfunction and BD. For instance, elevated cortisol can indirectly cause hypothyroidism by suppressing the HPT axis by inhibiting TRH from the hypothalamus and the release of TSH from the pituitary gland [33]. Additionally, cortisol inhibits the conversion of T4 to T3 in peripheral tissues by interfering with the enzyme 5’-deiodinase by decreasing its expression [33]. This results in lower levels of active thyroid hormone, which contributes to the suppression of the HPT axis [33]. Moreover, elevated cortisol can exacerbate mood symptoms implicated in BD. Chronic stress and dysregulation of the HPT axis may also contribute to mood instability and symptom severity in BD [32]. Additionally, alterations in thyroid hormone levels can impact mood, energy, and cognition in individuals with BD as hypothyroid symptoms may overlap with symptoms of depression in BD causing difficulty in the diagnosis and management of both conditions [32].

Findings from multiple research studies have sought to evaluate cortisol and its role in linking hypothyroidism and BD. One group of investigators retrospectively identified 41 studies that demonstrated BD being consistently associated with increased cortisol and ACTH, but not CRH [34]. Furthermore, another study measuring average cortisol concentrations in saliva and hair has found a twofold increase in patients with BD than in control populations [35]. Elevated cortisol may precipitate manic or depressive symptoms in nonpsychiatric patients, as frequently seen in patients with Cushing’s syndrome [36]. One investigation found elevated serum cortisol and hypothyroidism to be linked with increased rates of depressive episodes, anxious symptoms, and cognitive impairment [37]. The researchers additionally found that there was a notable increase in mood disorders in those with hypothyroidism in comparison to euthyroid controls [37]. These associations further support the claim that elevated levels of cortisol may provide insight into the relationship between thyroid dysfunction and BD. 

Individuals with bipolar disorder tend to exhibit a diminished stress response, as evidenced by a sibling study quantifying cortisol and alpha-amylase fluctuations in response to stressors [38]. The experimental group, comprising euthyroid patients with BD under pharmacological management, underwent stressful tasks akin to the Trier Social Stress Test (TSST) [38]. Salivary cortisol and alpha-amylase levels were measured at baseline, pre-task, and post-task, alongside subjective stress assessments. Compared to healthy controls, the experimental group displayed attenuated cortisol increases and elevated alpha-amylase levels post-task [38]. Moreover, abnormal hormonal response variations were more prominent in individuals with a history of recurrent bipolar episodes [38]. The study also cited literature suggesting a link between baseline cortisol levels and offspring of parents with BD, hinting at HPT axis dysfunction in BD [39]. A meta-analysis encompassing 42 studies utilizing diverse measures such as the Life Events and Difficulties Schedule (LEDS), Interview for Recent Life Events (IRLE), Social Readjustment Rating Scale (SRRS), Paykel Life Events Scale (PLES), BDI, and hospital admissions examined the impact of stressful life events on acute BD symptoms. The analysis revealed that patients with BD reported higher levels of stressful life events compared to individuals with other psychiatric conditions, yet no significant difference was observed in the effects of these events on the former [39]. These findings, although derived from distinct studies with varying populations, emphasize the significant role of stress in the onset and exacerbation of BD [39]. 

Inflammation and Immune Dysregulation

Chronic inflammation appears to be implicated in the pathogenesis of both BD and thyroid dysfunction, potentially providing a key link between the two conditions. Elevated levels of C-reactive protein, a marker of ongoing inflammation, have been observed in patients with BD during acute manic episodes [40]. Additionally, increased concentrations of pro-inflammatory cytokines such as interleukin 1, 2, 6, and TNF-a have been documented [41]. Furthermore, IL-6 levels were found to decrease following resolution of manic episodes in a separate study [41]. In hypothyroidism, diminished antioxidant levels exacerbate the chronic inflammatory state, while hyperthyroidism is associated with increased production of reactive oxygen species (ROS) [32]. Moreover, patients with schizophrenia (SZA) exhibited elevated plasma levels of peroxides and malondialdehyde, indicating oxidative stress [32]. This cycle of chronic inflammation leading to immune dysfunction, perpetuating further inflammation and structural damage, is discussed in detail within the neuroinflammation context in the subsequent section.

The relationship between autoimmune thyroiditis and BD necessitates further research to establish an association. For instance, a Danish study of 9,920 patients with BD investigated the precedence of autoimmune thyroiditis and thyrotoxicosis, although the results were not statistically significant [42]. However, conditions like rheumatoid arthritis, psoriasis, ulcerative colitis, autoimmune hepatitis, and Guillain-Barré syndrome were significant antecedents, indicating a potential contribution of immune dysfunction to BD's etiology [42]. Conversely, other epidemiological studies have discussed the prevalence of autoimmune conditions in the presence of BD, namely autoimmune thyroiditis, systemic lupus erythematosus, autoimmune hepatitis, multiple sclerosis, and rheumatoid arthritis [34]. Additionally, a study involving 51 monozygotic and dizygotic twins diagnosed with BD, along with 35 control twins, revealed a higher prevalence of thyroid peroxidase antibodies (TPO-Abs) in the diagnosed group compared to controls [43]. Particularly, bipolar female twins exhibited a higher correlation with autoimmune thyroid dysfunction than their male counterparts, though this observation requires further investigation due to the skewed gender distribution in this sample [43]. Another study found a robust association between TPO-Abs and Tg-antibodies in rapid-cycling BD, with TPO-Abs showing stronger statistical significance [44]. While the shared pathways of chronic inflammation between BD and autoimmune thyroiditis continue to be examined, there have been recent clinical trials aimed at investigating the therapeutic effects of anti-inflammatory medication in those with BD. The use of N-acetyl-cysteine was found to have a clinical and significant difference in minimizing the severity of depressive symptoms in those with BD [34]. However, the effects of non-steroidal anti-inflammatory drugs (NSAIDs) during manic and hypomanic episodes have yielded mixed results and thus, it is unclear if NSAIDs help decrease these mood symptoms [34]. With a better understanding of chronic inflammation and these autoimmune processes as they relate to BD, we can make greater advancements in targeted pharmacologic treatment.

Neuroinflammation and Neuroimaging 

A recent systematic review compiled data that demonstrated volumetric changes in various regions of the brain, specifically those involved in emotional regulation and affect [45]. These abnormalities were noted in both functional and structural MRI scans and were associated with inflammatory markers, thereby strengthening the hypothesis that pro-inflammatory mechanisms play a pivotal role not only in the pathogenesis of BD but also in the exacerbation of symptoms [45]. This central and peripheral inflammation can lead to progressive impairment and higher stress levels that cyclically perpetuate the chronic inflammatory state [45]. 

Furthermore, neuroimaging trends observed in another study of BD cases yielded gray matter reduction in prefrontal and limbic regions, lateral ventricle enlargement, deep white matter hyperintensities, and a hyperactive amygdala, striatum, and thalamus [42]. Glutamate and NMDA receptor function, resulting in excitotoxicity of nervous tissue via the arachidonic acid cascade, has also been observed in the setting of neuroinflammation [42]. Arachidonic acid, a component of cellular membrane phospholipids, releases pro-inflammatory products when activated by phospholipase A2 as part of normal neurotransmission [25]. This cascade is upregulated by astrocytes in the presence of excess glutamate leading to neuronal damage [25]. Although some studies suggest lithium demonstrated antioxidant effects and was therefore neuroprotective, in animal models, prolonged exposure to other antipsychotics like quetiapine and olanzapine was shown to increase oxidative damage to the brain with subsequent loss of astrocytes [25,42].

Genetic insights: common factors influencing thyroid dysfunction and bipolar disorder onset

Autoimmune Thyroiditis and Bipolar Disorder

Recent research has explored the connection between autoimmune thyroiditis and BD, identified by TPO-Abs, revealing potentially shared genetic foundations and clinical implications. Researchers investigated this association by analyzing blood samples from 22 monozygotic and 29 dizygotic bipolar twins, along with 35 healthy control twins, for TPO-Abs [43]. TPO-Abs were found in 27% of twins with BD, with similar rates in their siblings and twins without BD [43]. Discordant twins had higher rates of autoimmune thyroiditis than healthy controls, suggesting a link between autoimmune thyroiditis, BD, and genetic predisposition [43]. This finding may serve as an endophenotype indicating genetic vulnerability to BD [43]. A study conducted by Snijders resulted in stable TPO-Abs levels over six to 12 years in patients with BD, suggesting these antibodies are a trait marker for the disorder [46]. However, according to the investigators, routine TPO-Abs screening in BD patients or their relatives is not recommended unless specific thyroid-related symptoms or abnormal hormone levels are present due to a lack of evidence to suggest otherwise [46]. Another study investigated the prevalence of autoimmune thyroiditis in offspring of parents with BD. A total of 140 offspring were evaluated for 55 months, with researchers conducting psychiatric assessments and blood tests for TPO-Abs and serum TSH levels [47]. Results exhibited higher TPO-Abs prevalence in female offspring of parents with BD, with some exhibiting thyroid failure [47]. Despite no observable increase in affective disorders or psychopathologies in TPO-Ab-positive offspring, the study suggests an independent vulnerability to autoimmune thyroiditis among offspring of BD parents [47]. 

Genome-Wide Association Studies

​​Genome-wide association studies (GWAS) have identified genetic variations associated with both thyroid function and BD, focusing on genes regulating thyroid hormones and those linked to BD susceptibility. In 2008, Baum conducted a GWAS to explore genetic markers in BD patients, finding evidence of genetic association at multiple loci [48]. While no major gene abnormalities were observed, individuals with the identified loci showed a significantly higher number of risk alleles, particularly at the DGKH locus [48]. This suggests a polygenic nature of BD, with risk influenced by the accumulation of dominant risk alleles rather than major gene effects [48]. Furthermore, GWAS research has revealed that both BD and thyroid dysfunction involve genes associated with immune function and inflammation, such as the human leukocyte antigen (HLA) genes [49]. Likewise, polygenic risk scores (PRS), derived from GWAS data, have offered a valuable tool for assessing genetic susceptibility to both BD and thyroid dysfunction. By combining information from numerous single nucleotide polymorphisms (SNPs) via GWAS, it is possible to identify individuals at high risk for these conditions based on their genetic variants [49]. 

Soheili-Nezhad conducted a comprehensive analysis of phenotypic and genetic data obtained from GWAS to further examine potential associations between thyroid function and psychiatric disorders [50]. Investigators found significant connections between symptoms of hypothyroidism and mood disorders, particularly BD and pinpointed loci within 6p22.1 and 6p22.2 as potential mediators of genetic influence between thyroid and bipolar disorders [50]. Moreover, the MHC region was implicated as this locus has been observed across multiple autoimmune-related clinical conditions favoring an association mediated through pathways involved in disease pathogenesis akin to autoimmunity over thyroid hormones [50].

Other psychiatric disorders, specifically SZA, have been analyzed in comparison to BD in the setting of GWAS. This association has piqued interest due to the overlap of BD and schizophrenic symptoms such as delusions, irritability, and disorganization. For instance, another GWAS phenotyped 2,586 BD patients undergoing lithium therapy between 2008 and 2013 by using a PRS approach to analyzing genome-wide SNPs for SZA risk [51]. This study revealed a negative correlation between the genetic predisposition for SZA risk variants and the responsiveness to lithium treatment among individuals with bipolar affective disorder [51]. Genetic variations within the HLA antigen region and the antigen presentation pathway shed light on the molecular mechanisms underlying both SZA and the response to lithium treatment [51]. Evaluating the polygenic burden of SZA risk variants, along with clinical information, may aid in predicting the likelihood of lithium treatment response in patients with bipolar affective disorder [52]. Additional investigation employing GWAS is necessary to clarify the complex connection between thyroid dysfunction and BD.

Impact of Family History on Susceptibility of Bipolar Disorder and Thyroid Dysfunction

Recognizing the significance of family history in BD and thyroid dysfunction, particularly in the context of genetic susceptibility, sheds light on the shared genetic basis of these conditions. Family history serves as a crucial indicator of genetic predisposition to BD and thyroid dysfunction. It has been demonstrated that BD has a high heritability rate and significant genetic overlap with other psychiatric disorders [53]. Similarly, genetic susceptibility plays a role in thyroid dysfunction, where variations in genes associated with immune response and inflammation contribute to its development [53]. 

Understanding the genetic correlations between BD and other psychiatric disorders, such as SZA and MDD, provides insights into the shared genetic architecture underlying these conditions [54]. For example, studies have demonstrated significant genetic overlap between BD and SZA, highlighting the importance of considering familial history and genetic predisposition when assessing risk for these disorders [54]. A recent study employed Mendelian randomization to explore the causal relationship between genetic factors affecting thyroid function and the risk of BD [55]. Using genetic variants associated with thyroid traits as instrumental variables, they found significant genetic links between the two [54]. Specifically, a genetic predisposition to higher T4 levels was causally associated with an increased BD risk, suggesting thyroid hormone dysregulation may play a role in BD development [55]. Bidirectional causality was observed; while elevated T4 levels increased BD risk, genetic susceptibility to BD also influenced thyroid traits [55]. By interpreting the shared genetic foundations of these conditions and their correlations with other psychiatric and non-psychiatric disorders, clinicians can improve early prevention strategies and develop more personalized treatment approaches based on individual genetic risk profiles.

Optimizing treatment: enhancing management for thyroid dysfunction in bipolar disorder

Research has demonstrated that using thyroid hormones as an adjunctive therapy holds more promise for affective disorders [56]. It has been observed that higher doses of L-thyroxine (L-T4) are necessary to achieve a therapeutic effect in patients with BD compared to those with primary thyroid disorders [56]. Despite concerns about adverse effects, patients with affective disorders generally tolerate these higher doses without major issues [56].

Numerous investigations have been conducted to examine the impact of thyroid hormone treatment on BD management. In a randomized, placebo-controlled double-blinded trial, Bauer explored the effectiveness of high-dose L-T4 in mitigating depressive symptoms in euthyroid individuals with bipolar depression [57]. Twenty-five bipolar depression patients resistant to mood stabilizers and/or antidepressants were randomly assigned to receive either L-T4 or placebo for six weeks [57]. PET scans revealed reduced metabolic activity in the thalamus and ventral striatum after six weeks of L-T4 treatment, indicating improved depressive symptoms [57].

In another study, a 7-week open-label investigation assessed the adjunctive use of high-dose L-T4 in women with treatment-resistant bipolar depression [53]. Significant decreases in depression scores were observed, alongside PET scan findings showing alterations in brain metabolism, particularly in prefrontal, subcortical, and limbic regions, contributing to mood improvement in patients with refractory bipolar depression [53]. Furthermore, in Bauer’s 2005 and 2015 studies, the effectiveness of L-T4 treatment was evaluated by assessing changes in depression scales from baseline scores [53,58]. In the 2005 study, the primary measure was the Hamilton Rating Scale for Depression (HRSD21), with the BDI as a secondary measure [53]. Full remission was defined as a 50% reduction in HRSD21 score with a final score of <7.62. Using the t-test (P<0.001), significant improvements in depression scores from baseline with L-T4 treatment were observed [53]. In the 2015 study, treatment responses were evaluated based on changes in the Hamilton Rating Scale for Depression (HamD17) [58]. A response to treatment was defined as having post-treatment scores reduced by ≥50%. L-T4 treatment responders showed a mean decrease of 67%, with a clinically significant difference in post-treatment values compared to the placebo group [58]. These findings are summarized in Table 3.

**Table 3:** Behavioral Measures Before and After LT4 Treatment in Patients With Bipolar Depression The table summarizes behavioral measures observed in patients with bipolar depression before and after LT4 treatment. Data is adapted from clinical observations and assessments conducted by Bauer et al. in 2015 and Bauer et al. in 2005 [53,58]. BDI, Beck Depression Inventory

	Pretreatment (Baseline)	Post-treatment
Hamilton Rating Scale for Depression (HRSD21); LT4 320 mcg	23.2±5.0	6.0±3.3
BDI; LT4, 320 mcg	33.4±9.8	11.6±8.7
Hamilton Rating Scale for Depression (HamD17)		
LT4, 300 mcg	21.7±3.2	12.6
Placebo	20.3±6.3	16.3

It is theorized that using thyroid hormone to restore patients with BD to a euthyroid state can effectively reduce neuropsychiatric symptoms [53]. Therefore, it is logical to consider employing thyroid hormone to alleviate symptoms of BD in the setting of thyroid dysfunction. Although monotherapy with thyroid hormone has shown limited success, adjunctive treatment with supraphysiological levels of L-T4 alongside antidepressants can lead to a reduction in depressive symptoms and improved brain metabolism, as evidenced by studies mentioned above [53,58].

## Conclusions

The review highlights the bidirectional relationship between thyroid dysfunction and BD, focusing on neurochemical dynamics, including neurotransmitter imbalances and neuroinflammatory processes, and emphasizing the role of the HPT axis in mood stabilization. Genetic factors are also discussed, revealing potentially shared genes and molecular pathways. Integrated treatment approaches are stressed, emphasizing the importance of understanding this interplay for improving outcomes in affected individuals. Ongoing research into shared genetic and epigenetic factors is vital for understanding the connection between thyroid dysfunction and BD and guiding personalized treatments to maximize therapeutic effects, improve prognosis, and reduce exacerbation of BD symptoms. Likewise, standardized screening protocols for thyroid dysfunction in BD patients can enable earlier and more precise intervention and tailored treatment plans, enhancing a more holistic approach via care coordination between endocrinologists and psychiatrists.
